# Bloody Evidence: The Validity of Glycophorin A in the Determination of Wound Vitality—A Systematic Review of the Literature

**DOI:** 10.3390/ijms26115308

**Published:** 2025-05-31

**Authors:** Donato Morena, Anna Maria Manta, Alessandro Santurro, Matteo Scopetti, Emanuela Turillazzi, Vittorio Fineschi

**Affiliations:** 1 Department of Anatomical, Histological, Forensic and Orthopedic Sciences, Sapienza University of Rome, 00161 Rome, Italy; donato.morena@uniroma1.it (D.M.); annamaria.manta@uniroma1.it (A.M.M.); 2 Department of Medicine, Surgery and Dentistry “Schola Medica Salernitana”, University of Salerno, 84081 Baronissi, Italy; asanturro@unisa.it; 3 Department of Medical Surgical Sciences and Translational Medicine, Sapienza University of Rome, 00189 Rome, Italy; matteo.scopetti@uniroma1.it; 4 Department of Surgical Pathology, Medical, Molecular and Critical Area, University of Pisa, 56126 Pisa, Italy; emanuela.turillazzi@unipi.it

**Keywords:** forensic medicine, forensic pathology, glycophorins, wounds and injuries, immunochemistry, autopsy

## Abstract

In forensic pathology, glycophorin A (GPA) presence in tissues has been studied through anti-GPA monoclonal antibodies with immunohistochemical techniques. The use of anti-GPA in distinguishing ante- from postmortem lesions, particularly in advanced stages of decomposition, is explored in this study. This systematic review assesses the current use of glycophorins, focusing on their application in forensic investigations to detect vital hemorrhagic infiltration. A total of 799 studies were identified, and after screening, 16 studies were included, encompassing case reports, experimental studies, and case-control analyses. The data extracted from these studies highlighted the diagnostic accuracy of GPA immunohistochemical staining in forensic cases. Of the 50 specimens analyzed, 48 were stained with anti-GPA serum and 2 with anti-glycophorin C (GPC) serum. The results showed that GPA staining was significantly more reliable in detecting vital hemorrhage compared to routine histology, which had a diagnostic accuracy of only 66%. Moreover, in an experimental study, GPA positivity was observed in 72.2% of bruises and vital tissues, even in highly decomposed specimens. The study further emphasized the robustness of GPA in distinguishing ante- from postmortem injuries, with particular relevance for cases involving significant decomposition. Overall, GPA’s high sensitivity in detecting vital hemorrhage, especially in decomposed bodies, underscores its potential as a reliable tool in forensic pathology. Despite some limitations due to the small number of studies, the evidence supports the inclusion of anti-GPA antibodies in routine forensic examinations, potentially enhancing the accuracy of wound vitality assessments in criminal investigations.

## 1. Introduction

Glycophorins are a family of glycoproteins that play a crucial role in the structural integrity and functionality of red blood cells (RBCs). They are integral membrane proteins predominantly found in the erythrocyte membrane and are known for their heavily glycosylated extracellular domains. The main members of the glycophorin family include glycophorin A (GPA), glycophorin B (GPB), glycophorin C (GPC), and glycophorin D (GPD). These proteins have distinct structural and functional characteristics, contributing to various physiological and immunological processes.

Glycophorin A is the most abundant glycophorin, with its primary function being the preservation of erythrocyte shape and deformability. GPA’s extracellular domain is heavily glycosylated, providing a negative charge that helps prevent RBCs aggregation and adhesion to endothelial cells [1]. Additionally, GPA serves as a receptor for the malaria parasite Plasmodium falciparum, which binds to it to invade RBCs, and, while not exclusively, it may also function as a receptor for certain viruses that recognize sialic acid residues, such as influenza A and B, group C rotavirus, reoviruses, and possibly hepatitis A virus, although evidence for the latter is limited [2,3,4].

Research into glycophorins continues to provide valuable insights into their functions and potential applications in clinical and therapeutic contexts, as well as in forensic investigations.

Anti-human GPA (anti-GPA) monoclonal antibodies are commonly used in human blood detection due to their specificity to RBCs and resistance to environmental degradation [5,6]. They are valuable markers in identifying bloodstains and determining the presence of human blood in forensic samples, even after extended exposure to harsh conditions. Detection of GPA aids in distinguishing between human and animal blood, enhancing the accuracy of forensic analyses [7].

Moreover, anti-GPA can be used in immunohistochemical staining in postmortem examinations to highlight RBCs presence in tissues [8]. This marker is particularly valuable since it can be used on different types of specimens and has proven to resist postmortem decomposition [9].

While macroscopically evident hemorrhagic infiltration of the tissue can be considered suggestive of wound vitality, anti-GPA antibodies could aid in the differential diagnosis between ante- and postmortem lesions. This is particularly relevant since the recognition of the vitality of the wound, together with the determination of the cause of death, remains one of the major challenges in forensic practice despite the technological advances in the field [10,11].

Trichrome staining including Azan’s method or the Van Gieson elastic are also used in many forensic laboratories to allow, on corpses in an advanced stage of putrefaction, better visualization of both tissue and the cellular component, such as erythrocytes. Immunohistochemical methods, however, have largely replaced classical staining and, especially antibodies against glycophorins, spectrin, and hemoglobin, can give good results. The search is for spectrin beta chain in human erythrocytes, but since GPA, also known as CD235a or erythrocyte marker, is the main intrinsic membrane protein of the erythrocyte, our experience has always been devoted to its search. Microscopic techniques such as laser confocal microscopy can also give good results, enabling higher resolution and three-dimensional image reconstruction.

Studies on this matter emphasize that the detection of GPA is highly valuable for both bone structures and soft tissues, particularly when bodies are discovered in an advanced state of decomposition [8,9]. In the context of hemorrhagic events, the presence of erythrocyte extravasation is indicative of a vital (antemortem) physiological response. A negative immunohistochemical reaction for GPA indicates the absence of RBCs in the examined tissue, supporting a postmortem origin of the wound. Conversely, a positive GPA result, demonstrating the presence of RBCs located outside vascular structures, confirms hemorrhage consistent with an antemortem injury and suggests that the wound occurred while blood circulation was still active.

Currently, the use of anti-GPA antibodies remains experimental in forensic pathology. However, as previously observed, it has shown promise for application in various tissue types.

Nonetheless, the number of studies on reliable markers of wound vitality in decomposed bodies remains limited, whereas diagnosing wounds in non-decomposed skin is generally much easier than in putrefied tissues. Furthermore, the applicability and reproducibility of these techniques are often uncertain, raising doubts about the validity of these markers and diminishing their scientific value in criminal judicial settings.

Therefore, an overview of the current state of knowledge regarding the use of the glycophorin family in forensic practice is provided through a systematic review of the literature. In addition to a detailed analysis of existing studies, a recently disclosed Italian legal case is briefly presented involving the deceased body of a male subject that underwent two separate postmortem examinations more than two decades apart. New evidence obtained through the application of immunohistochemical techniques using GPA led to the reopening of a case that had been legally closed as a suicide years earlier. This ultimately resulted in a diagnosis of homicide and the conviction of the suspected perpetrator.

Finally, the ultimate goal of this study is to provide new scientific and judicial evidence supporting the validation of anti-GPA as a reliable marker of vital hemorrhage. This technique could become routine, particularly in cases where determining the nature of a lesion is crucial in forensic criminal investigations.

## 2. Methodology

A search for published studies was conducted in PubMed, Scopus, Web of Science, and Google Scholar (for search strategy, see Appendix SA, for the screening process, Appendix SB in Supplement S1). Google Scholar was manually inspected, where we entered the same search keywords used for searches on other databases. As a first step, results from multiple databases were merged, and duplicate records were automatically removed. In the second step, additional duplicates were manually eliminated through title screening. Eligibility assessment was then performed based on the abstracts of the identified studies. Papers that passed this initial screening process underwent a more comprehensive assessment for potential inclusion in our study, which involved thoroughly examining the full text. The inclusion criteria encompassed all experimental studies, case studies, and case series that focused on the analysis of glycophorins in histological samples. The exclusion criteria included non-English language and non-human studies. Two independent raters (D.M. and A.M.M.) extracted relevant study characteristics and outcome measures data. Discrepancies during screening and data extraction were resolved through discussion, with arbitration by senior review authors (E.T. and V.F.) when necessary, particularly concerning the relevance of the data to our study. Emerging reviews and the reference lists of retrieved papers were also manually searched by two investigators (D.M. and A.M.M.). This systematic review was designed and carried out according to the Preferred Reporting Items for Systematic Reviews and Meta Analyses (PRISMA) guidelines [12,13]. All authors of studies from which unpublished data could be obtained were contacted via email twice. When relevant information required for inclusion in our study could not be obtained from the authors, a meticulous review of the studies was conducted through a conference between raters (D.M. and A.M.M.) and the senior authors (E.T. and V.F.).

## 3. Data Extraction

**Figure 1:**
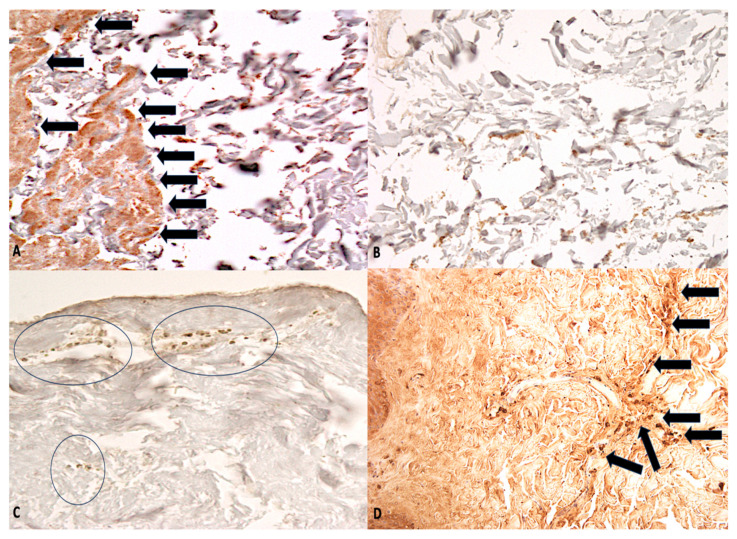
(**A**,**B**) Lung in advanced putrefaction: in (**A**), well-defined immunohistochemical reaction to GPA (arrows) is evident, in comparison with the control sample of similarly putrefied lung (**B**) (×80). (**C**,**D**) Skin showing GPA positivity in papillary layer of dermis (circles) (**A**) and in reticular layer of dermis (arrows) (**B**) (viable ligature mark) (×60).

**Figure 2:**
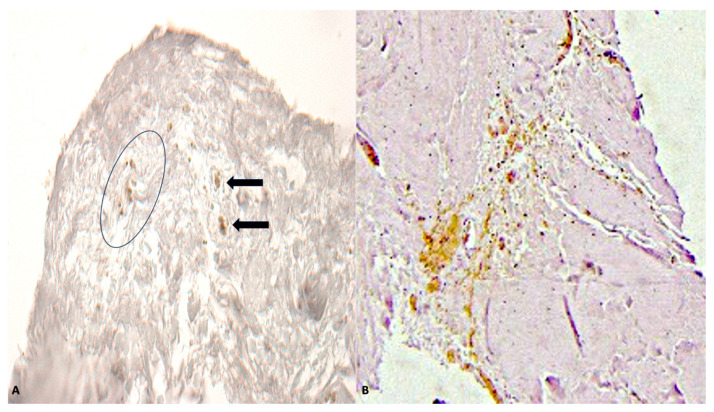
(**A**) Skin in subject in advanced putrefaction showing GPA positivity (circle) in deep dermal layer (arrows) (bruises) (×80). (**B**) Nervous tissue with GPA positivity (in brown) indicating hemorrhage in deep layers (×80).

A standardized form was used to extract data from the included studies, which involved case reports, case series, and case-control studies, to summarize the results. The extracted information included the year of publication; author(s); type of marker analyzed (i.e., GPA, GPC); case characteristics such as age, sex, cause of death, manner of death, and presence of stress factors; vital time; postmortem interval (PMI); intensity of putrefaction; type of lesion; tissue type; immunohistochemistry results; presence of microscopic hemorrhagic infiltration visible with hematoxylin and eosin (H&E) staining; concordance between glycophorins and H&E staining; and concordance between positivity/negativity for glycophorins and the confirmed presence/absence of blood material (Figure 1 and Figure 2). Extraction was independently conducted by two reviewers (D.M. and A.M.M.) in duplicate. Senior authors (E.T. and V.F.) were consulted when needed.

## 4. Results

Initially, 799 studies were found (PubMed = 213; Scopus = 386, WoS = 200), from which 201 duplicates were automatically removed and an additional 139 were manually eliminated (n = 459). The title and abstract review allowed for the selection of 50 potentially eligible contributions. Reference list screening and a supplementary search on Google Scholar did not yield any further potentially eligible studies.

**Figure 3:**
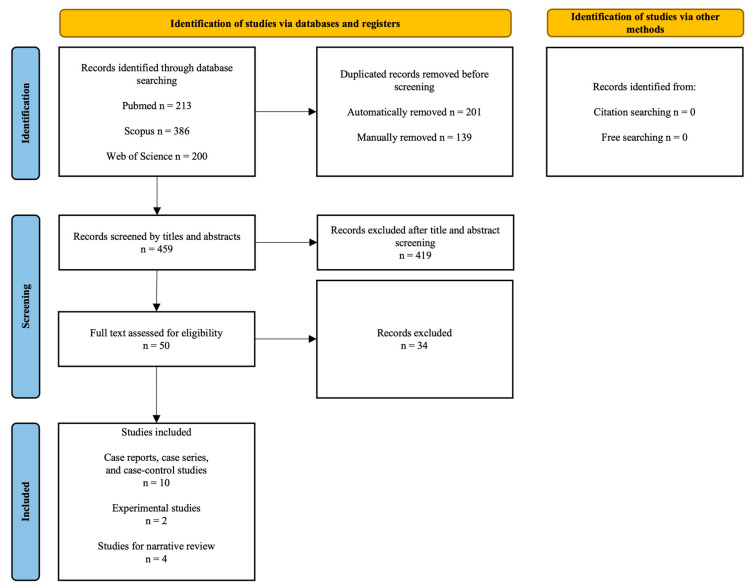
PRISMA flow chart of the study selection process.

Figure 3 shows the study selection procedure.

After reviewing the full content of the papers (for the list of the included and excluded studies, with motivation, see Appendix SC in Supplement S1), the following studies were included:

- Ten case reports, case series, and case-control studies from which appropriate data were extracted and which are summarized in Table 1 [6,9,10,14,15,16,17,18,19,20];
- Two experimental studies [8,21];
- Four articles, which were included in the narrative overview [22,23,24,25].

Risk of bias was assessed using JBI^®^’s critical appraisal tools (e.g., JBI Critical Appraisal Checklist for Case Reports, JBI^®^ Critical Appraisal Checklist for Case Series, and JBI^®^ Critical Appraisal Checklist for Case Control Studies [26,27]; see Appendix SD in Supplement S1). All included studies demonstrated a fair methodological quality.

Table 1 summarizes the results of the case review in the literature. In some instances, more than one sample was obtained.

**Table 1:** Samples in which anti-glycophorin antibodies were used to detect RBCs.

Year	Author	Marker	Sex/Age (Years)	Cause of Death	Manner of Death and/or Presence of Stress Factors	Vital Time	PMI	Intensity of Putrefaction	Type of Lesion	Tissue (Site)	IHC Result (C/NC)	Presence of Hemorrhagic Blood Elements on Histology	Underlying Diseases
**Cases**													
2021	Baldari et al.—case “1” [9]	GPA	F/57	Head injury	Homicide (attempted sexual assault)	n.r.	65 days	Severe	Putrefactive injured area	Bone (cranial)	Pos. (C)	Neg.	n.r.
2021	Baldari et al.—case “2”	GPA	M/30	n.s.	Accident (n.s).	n.r.	70 days	Severe	Putrefactive injured area	Bone (vertebral)	Pos. (C)	Neg.	n.r.
2021	Baldari et al.—case “3”	GPA	F/39	Asphyxiation	Homicide (n.s.)	n.r.	2 days	Mild	Putrefactive injured area	Bone (mandible)	Pos. (C)	Pos.	n.r.
2021	Baldari et al.—case “4”	GPA	F/46	Asphyxiation	Homicide (strangulation)	n.r.	181 days	Severe	Putrefactive injured area	Soft tissue (larynx)	Pos. (C)	Neg.	n.r.
2021	Baldari et al.—case “5 a”	GPA	F/37	Asphyxiation	Homicide	n.r.	85 days	Severe	Putrefactive injured area	Skin (neck)	Pos. (C)	Neg.	n.r.
2021	Baldari et al.—case “5 b”	GPA	F/37	a.p.a.	a.p.a.	a.p.a.	a.p.a.	a.p.a.	a.p.a.	Bone (rib)	Pos. (C)	Neg.	n.r.
2021	Baldari et al.—case “6”	GPA	F/3 months	Massive brain edema (due to head injury)	Homicide	n.r.	7 days	Mild	Putrefactive injured area	Soft tissue (retina)	Pos. (C)	Pos.	n.r.
2023	Di Fazio et al.—case “3” [14]	GPA	unc./82	Bleeding—heart failure; overdose of enoxaparin sodium	Homicide	n.r.	621 days	Severe	Areas with hemorrhagic infiltration at gross examination	Skin (n.r.)	Pos. (C)	Pos.	Heart dilation, with a pacemaker and mitral valve prosthesis, moderate aortic insufficiency, chronic atrial fibrillation under anticoagulant therapy, and chronic pancreatitis
2023	Di Fazio et al.—case “4 a”	GPA	unc./77	Bleeding—heart failure; overdose of enoxaparin sodium	Homicide	n.r.	697 days	Severe	Areas with hemorrhagic infiltration at gross examination	Skin (n.r.)	Pos. (C)	Pos.	Hip and knee implants, non-Hodgkin’s lymphoma, former smoker
2023	Di Fazio et al.—case “4 b”	GPA	unc./77	a.p.a.	a.p.a.	n.r.	697 days	Severe	Areas with hemorrhagic infiltration at gross examination	Muscle (n.r.)	Pos. (C)	Pos.	a.p.a.
2023	Di Fazio et al.—case “5 a”	GPA	unc./88	Bleeding—chronic subdural hematoma with recent bleeding; overdose of enoxaparin sodium	Homicide	n.r.	742 days	Severe	Areas with hemorrhagic infiltration at gross examination	Skin (n.r.)	Pos. (C)	Pos.	Severe cerebrovascular and degenerative dementia with psychosis, diabetes mellitus, chronic atrial fibrillation under anticoagulant therapy, chronic HCV and HBV infection
2023	Di Fazio et al.—case “5 b”	GPA	unc./88	a.p.a.	a.p.a.	n.r.	742 days	Severe	Areas with hemorrhagic infiltration at gross examination	Skin (n.r.)	Pos. (C)	Pos.	a.p.a.
2023	Di Fazio et al.—case “5 c”	GPA	unc./88	a.p.a.	a.p.a.	n.r.	742 days	Severe	Areas with hemorrhagic infiltration at gross examination	Skin (n.r.)	Pos. (C)	Pos.	a.p.a.
2023	Di Fazio et al.—case “6 a”	GPA	unc./88	Bleeding—pulmonary embolism and sepsis caused by a pacemaker infection; overdose of enoxaparin sodium	Homicide	n.r.	878 days	Severe	Areas with hemorrhagic infiltration at gross examination	Skin (n.r.)	Pos. (C)	Pos.	Bronchial asthma, ankylosis of the left upper limb, diabetes mellitus type 2, recent implantation of pacemaker
2023	Di Fazio et al.—case “6 b”	GPA	unc./88	a.p.a.	a.p.a.	n.r.	878 days	Severe	Areas with hemorrhagic infiltration at gross examination	Muscle (n.r.)	Pos. (C)	Pos.	a.p.a.
2023	Di Fazio et al.—case “6 c”	GPA	unc./88	a.p.a.	a.p.a.	n.r.	878 days	Severe	Areas with hemorrhagic infiltration at gross examination	Skin (n.r.)	Pos. (C)	Pos.	a.p.a.
2023	Di Fazio et al.—case “6 d”	GPA	unc./88	a.p.a.	a.p.a.	n.r.	878 days	Severe	Areas with hemorrhagic infiltration at gross examination	Skin (n.r.)	Pos. (C)	Pos.	a.p.a.
2023	Di Fazio et al.—case “6 e”	GPA	unc./88	a.p.a.	a.p.a.	n.r.	878 days	Severe	Areas with hemorrhagic infiltration at gross examination	Muscle (n.r.)	Pos. (C)	Pos.	a.p.a.
2015	Plowey and Egbert—case “1” [15]	GPC	M/7 weeks	unc.	Blunt force trauma to the thorax	n.r.	20 hours	None	No retinal hemorrhages on gross examination.	Soft tissue (retina)	Neg. (C) (intravascular)	Neg.	n.r.
2015	Plowey and Egbert—case “2”	GPC	F/7 weeks	unc.	unc.	n.r.	unc.	unc.	Multifocal retinal hemorrhages	Soft tissue (retina)	Pos. (C)	Pos.	n.r.
2013	Ambrosetti et al.—case “1 a” [16]	GPA	F/11	Sudden cardiac death	Natural	n.r.	n.r.	none	Hypostasis	Soft tissue (hymen)	Neg. (C)	Neg.	Brugada phenotype at DNA test
2013	Ambrosetti et al.—case “1 b”	GPA	F/11	a.p.a.	a.p.a.	n.r.	n.r.	none	Hypostasis	Soft tissue (navicular fossa)	Neg. (C)	Neg.	a.p.a.
2013	Ambrosetti et al.—case “1 c”	GPA	F/11	a.p.a.	a.p.a.	n.r.	n.r.	none	Any	Soft tissue (anus)	Neg. (C)	Neg.	a.p.a.
2021	Bertozzi et al. —case“1” [17]	GPA	n.r.	Gunshot	Suicide	n.r.	24–36 hours	Severe	Lacerated wound	Skin (n.r.)	Pos. (C)	unc.	n.r.
2021	Bertozzi et al.—case“2”	GPA	n.r.	Strangulation	Homicide	n.r.	3–6 days	Severe	Injured area	Skin (n.r.)	Pos. (C)	unc.	n.r.
2021	Bertozzi et al.—case“3”	GPA	n.r.	Hanging	Suicide	n.r.	3–6 days	Severe	Injured area	Skin (n.r.)	Pos. (C)	unc.	n.r.
2021	Bertozzi et al.—case“4”	GPA	n.r.	Slaughtering	unc. (probably suicide)	n.r.	7–10 days	Severe	Incised wound	Skin (n.r.)	Pos. (C)	unc.	n.r.
2021	Bertozzi et al.—case“5”	GPA	n.r.	Massive fracture head injury	unc. (probably accident)	n.r.	7–10 days	Severe	Lacerated wound	Skin (n.r.)	Pos. (C)	unc.	n.r.
2021	Bertozzi et al.—case“6”	GPA	n.r.	Burst head injury	unc. (probably homicide)	n.r.	10–15 days	Severe	Lacerated wound	Skin (n.r.)	Pos. (C)	unc.	n.r.
2021	Bertozzi et al.—case“7”	GPA	n.r.	Slaughtering	unc. (probably homicide)	n.r.	10–15 days	Severe	Incised wound of the neck	Skin (n.r.)	Pos. (C)	unc.	n.r.
2010	Cattaneo et al.—case “2” [18]	GPA	n.r.	n.r.	n.r.	34 minutes	n.r.	n.r.	Tibia fracture	Dry bone	Pos. (C)	Pos.	n.r.
2010	Cattaneo et al.—case “3”	GPA	n.r.	n.r.	n.r.	26 days	n.r.	n.r.	Cranium fracture	Dry bone	Pos. (C)	Pos.	n.r.
2010	Cattaneo et al.—case “6”	GPA	n.r.	n.r.	n.r.	8.5 hours	n.r.	n.r.	Rib fracture	Dry bone	Pos. (C)	Pos.	n.r.
2021	Crudele et al. [26]	GPA	M/63	Asphyxiation by hanging	Suicide	n.r.	180 hours	None	Intimal injury of carotid artery	Soft tissue (carotid wall)	Pos. (C)	unc. (H&E Neg., MT Pos.)	n.r.
2015	Maiese et al. [19]	GPA	M/28	Crush asphyxia	Accidental	few minutes	unc.	None	Imprint abrasion	Skin	Pos. (C)	n.r.	Fatty liver, heavy alcohol consumption
2021	Mazzarelli et al.—case “2 a” [20]	GPA	F/n.r.	Blunt force trauma and the body dismemberment	Probably homicide	n.r.	few months	Severe	Injured area (soft tissue residues around the lesioned bone)	Skin (scalp)	Pos. (C)	Neg.	n.r.
2021	Mazzarelli et al.—case “2 b”	GPA	F/n.r.	a.p.a.	a.p.a.	n.r.	few months	Severe	Injured area (soft tissue residues around the lesioned bone)	Skin (cervical)	Neg. (C)	Neg.	n.r.
2021	Mazzarelli et al.—case “2 c”	GPA	F/n.r.	a.p.a.	a.p.a.	n.r.	few months	Severe	Injured area (soft tissue residues around the lesioned bone)	Skin (axillary)	Neg. (C)	Neg.	n.r.
1997	Tabata and Morita—case “11” [6]	GPA	M/62	Asphyxiation	Drowning	n.r.	10 days	Moderate	Injured area	Skin (ankle)	Pos. (C)	unc.	n.r.
1997	Tabata and Morita—case “17”	GPA	M/unclear (20–39)	Asphyxiation	Drowning	n.r.	2–4 weeks	Severe	Injured area	Muscle (back)	Pos. (C)	Pos.	n.r.
**Controls**													
2021	Baldari et al.—control “1” [9]	GPA	F/9	Asphyxiation	Accidental (drowning)	n.r.	187 days	Severe	PM femur fracture	Bone (femur)	Neg. (C)	Neg.	n.r.
2021	Baldari et al.—control “2”	GPA	M/29	n.a. (exp.)	n.a.	n.r.	15 days	Mild	Putrefactive discoloration	Skin (unc.)	Neg. (C)	Neg.	n.r.
2021	Baldari et al.—control “3”	GPA	M/52	n.a. (exp.)	n.a.	n.r.	45 days	Severe	Putrefactive discoloration	Soft tissue (trachea)	Neg. (C)	Neg.	n.r.
2021	Bertozzi et al. control—“8” [17]	GPA	n.r.	Sudden cardiac death	Natural	n.r.	3–6 days	Severe	Skin losses by macro- and micro-fauna from the arms	Skin (n.r.)	Neg. (C)	unc.	n.r.
2015	Cappella et al.—control “5” [10]	GPA	n.r.	n.r.	n.r.	n.r.	48–72 hours	Severe	Parietal bone fragments	Bone	Pos. (C)	Pos.	n.r.
2015	Cappella et al.—control “6”	GPA	n.r.	n.r.	n.r.	n.r.	20 years	n.a.	Parietal bone fragments	Bone	Pos. (C)	Neg.	n.r.
2015	Cappella et al.—control “7”	GPA	n.r.	n.r.	n.r.	n.r.	20 years	n.a.	Parietal bone fragments	Bone	Pos. (C)	Neg.	n.r.
2015	Cappella et al.—control “8”	GPA	n.r.	n.r.	n.r.	n.r.	400 years	n.a.	Parietal bone fragments	Bone	Neg. (n.a.)	Neg.	n.r.
1997	Tabata and Morita—control “9” [6]	GPA	M/82	Acute cardiac failure	Natural	n.r.	7–10 days	Moderate	Hypostasis	Skin (hand dorsum)	Pos. (C)	Neg.	n.r.
1997	Tabata and Morita—control “20”	GPA	M/65	Asphyxiation	Drowning	n.r.	1–2 months	Severe	Control sample	Muscle (n.s.)	Pos. (C)	Neg.	n.r.

Abbreviations—a.p.a. = as per above; C = concordance between positivity/negativity and the presence/absence of a known hemorrhage; exp. = experimental; H&E = hematoxylin and eosin staining; IHC = immunohistochemical; MT = Masson’s trichrome staining; n.a. = not applicable; NC = non-concordance between positivity/negativity and the presence/absence of a known hemorrhage; Neg. = negative; n.r. = not reported; n.s. = not specified; PIA = putrefactive injured area; PM = postmortem; Pos. = positive.

The term “cases” refers to samples in which the presence of glycophorins was investigated without any prior assumptions or circumstantial evidence definitively indicating either a positive or negative result, particularly regarding the presence of hemorrhagic erythrocytic elements.

The term “controls” designates samples explicitly identified as such by the original authors, examined solely for comparative purposes with the reported cases. This group also included samples in which erythrocytic elements were confined to physiological structures.

A total of 50 specimens were extracted (42 cases and 8 controls), of which 48 were stained with anti-GPA serum and 2 with anti-GPC immunohistochemical techniques.

Among the samples, 11 were male, 12 were female, and 26 were classified as unclear or not specified. Age was reported in only 31 cases, ranging from a minimum of 2 weeks to a maximum of 88 years (median: 57 years).

**Figure 4:**
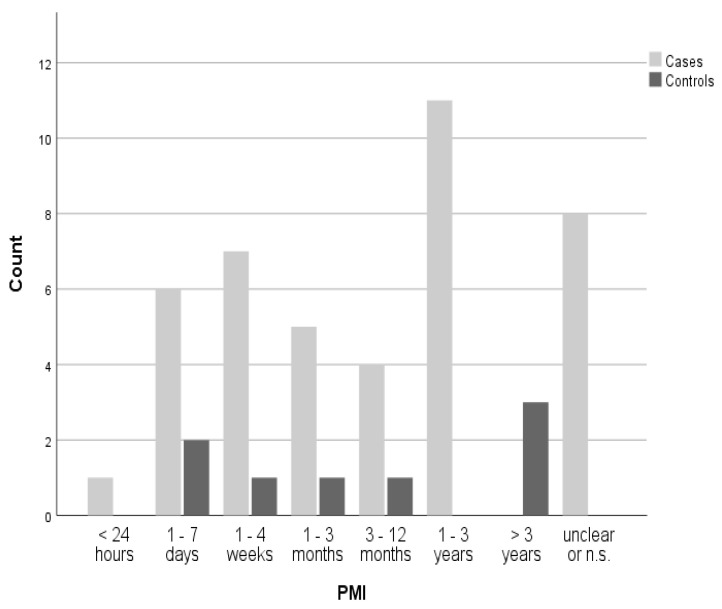
Postmortem interval (PMI) distribution among cases and controls. In cases of overlap, values were counted in the lower interval; n.s. = not specified.

The PMI was known in 42 cases, with the distribution between cases and controls shown in Figure 4.

**Figure 5:**
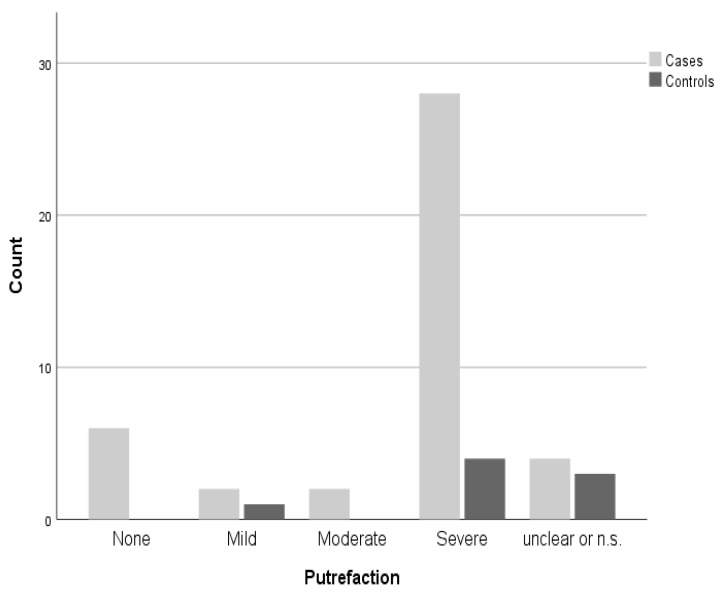
Distribution of putrefaction levels among cases and controls; n.s. = not specified.

The majority of the included cases (64%) exhibited severe levels of putrefaction. The levels of putrefaction were distributed as shown in Figure 5.

Differentiating between PMI and putrefaction was considered both useful and necessary, as the two conditions do not always correlate depending on the preservation state of the bodies or tissues.

## 5. Statistical Analysis

The diagnostic accuracy—defined as the concordance between GPA positivity/negativity and the presence/absence of a known hemorrhage —was complete, with only one case (Cappella et al.—control “8”) [10] in which it was not applicable. In contrast, the diagnostic accuracy using histology alone was 66%, with 18% of cases where hemorrhage, although present, was not detected, and 16% where the results were unclear. No statistically significant differences in histological accuracy were found between cases and controls (χ^2^ = 0.350, df = 2; *p* = 0.840).

## 6. Discussion

As previously mentioned, glycophorins have a wide range of applications in forensic investigations, with GPA appearing to be the most significant. However, as demonstrated by the present analysis, some authors have also utilized GPC for similar applications. Specifically, in the study of a microscopically visible inflammatory reaction, the use of the anti-GPC antibody helps to minimize the risk of counting leukocytes originating from passive extravasation in hemorrhagic infiltration while also facilitating the identification of wound margins [22].

Plowey and Egbert [15] also investigated the presence of hemorrhage using GPC instead of the GPA immunohistochemical technique. The rationale behind this choice is not explained by the authors, who employed this staining method to differentiate between Müller cell foot process swelling and nerve fiber layer hemorrhage. Specifically, GPC positively stained intravascular RBCs, with no evidence of hemorrhagic infiltration in the surrounding tissues, thereby indicating the absence of active hemorrhage. Additionally, it differentiated between hemorrhaged erythrocytes and adjacent swollen Müller cell foot processes, thus identifying retinal hemorrhages.

On the other hand, GPA has been used to confirm the presence of hemorrhagic infiltration, particularly in decomposed specimens, where the detection of intact RBCs is more challenging. Moreover, bruises have been analyzed and compared to intact skin using Western blotting, which demonstrated that GPA was only present in ecchymotic skin [25]. While the intensity and pattern of staining may vary depending on the degree of putrefaction, GPA deposits have been shown to retain positivity even after significant transformative changes [8]. Additionally, this technique is considered highly reliable, with studies comparing immunohistochemical findings to results obtained using different light sources for the macroscopic detection of bruises [23] and assessing changes in muscle fiber composition [24].

Furthermore, immunological analysis of GPA has been utilized to differentiate between putrefactive discoloration and bruises. No postmortem lividity exhibited a positive GPA reaction, suggesting that when present, a positive GPA reaction indicates a vital response to an insult. Conversely, GPA was detected in 13 out of 18 bruises (72.2%) using counter-immunoelectrophoresis and/or double immunodiffusion with anti-GPA serum rather than through direct microscopic visualization [21].

A consistent finding in the literature is that GPA immunohistochemistry frequently detects RBCs even when they are not identifiable by H&E staining.

For instance, Baldari et al. [9] reported GPA positivity in five out of seven samples lacking microscopically visible RBCs under H&E staining. In contrast, postmortem wounds produced in a controlled environment did not exhibit either hemorrhagic infiltration or GPA positivity. The authors postulated that while decomposition can affect the appearance of specimens and alter the structure of erythrocytes, GPA is highly resistant to postmortem decay and can be reliably detected in antemortem wounds.

Importantly, GPA remains diagnostically useful even in severely decomposed or macerated tissues. In the article published by Di Fazio et al. [14], the presence of hemorrhage in 14 deaths following inadequate heparin administration was investigated. Eight out of the fourteen bodies underwent exhumation, with a PMI ranging from 621 to 878 days. A total of 11 skin and soft tissue specimens were obtained, revealing the presence of RBCs with H&E staining, as well as GPA positivity in the same samples. The authors concluded that the vitality of the wounds was further supported by the strong GPA positivity.

Bertozzi et al. [17] selected seven cases and one control of decomposed bodies, where skin wounds (including gunshot wounds, sharp force injuries, ligature marks, and head injuries) were sampled and stained with a panel of immunohistochemical markers, including GPA. While the results of the H&E microscopic analysis are not reported by the authors, all the specimens stained positively for GPA. The authors concluded that while a multipanel of markers should be applied, the antibody found to be most reliable and recommended for routine use is anti-GPA.

Cattaneo et al. [18] extended this approach to bones. They analyzed the detection of microscopic markers of hemorrhage and wound healing on fractured bones with a known survival time. The specimens were initially macerated in tap water for at least 5 days and/or until all soft tissue was cleared from the bone. The samples were then prepared for H&E staining. Three out of six cases also underwent immunohistochemical analysis using GPA. Positivity was observed in all three cases, and the presence of blood clots was also evident in the H&E-stained samples. The study was the first to assess the feasibility of performing histopathological investigations on putrefied or macerated clean bone.

In addition to its resistance to decomposition, GPA plays a critical role in differentiating between ante- and postmortem injuries.

Ambrosetti et al. [16] reported a case of an 11-year-old female with suspicious reddish areas in the genital region. The hymen, fossa navicularis, and anus were sampled to investigate the presence of active hemorrhage and evaluate potential signs of sexual abuse. However, none of the sampled tissues exhibited RBCs, either with routine H&E staining or with anti-GPA serum. Based on these findings, the authors suggested that the nature of these pseudo-infiltrations was likely hypostatic, cautioning against the pitfall of misinterpreting these signs.

Crudele et al. [28] investigated the presence of microscopic intimal hemorrhage in Amussat’s sign in a non-decomposed body subjected to hanging. The carotid wall was sampled and stained with H&E, which did not reveal the presence of RBCs, and with Masson’s trichrome, which showed mild hemorrhagic infiltration. Additionally, anti-GPA immunohistochemistry was performed, highlighting the presence of multiple RBCs. The authors concluded that a thorough analysis of the ligature mark requires routine histopathological examination and immunohistochemistry to determine the vitality of the lesion.

In the case report by Maiese et al. [19], while primarily focusing on the usefulness of postmortem CT, a panel of markers, including GPA, was utilized. Immunohistochemistry with anti-GPA serum was performed on a skin abrasion to detect the presence of hemorrhage.

One of the cases presented by Mazzarelli et al. [20] utilized GPA to differentiate between ante- and postmortem wounds in a partially skeletonized body. Specifically, among the sampled skin wounds, one exhibited GPA positivity, while the others were negative. In contrast, H&E staining did not reveal the presence of RBCs. Consequently, it was concluded that only one of the wounds was vital.

A study by Tabata and Morita [6] investigated the usefulness of GPA as a marker of bleeding in autopsy cases with various degrees of decomposition, ranging from 12 h up to 2–3 months. Their findings demonstrated a positive reaction confined to blood vessels in intact skin, whereas in bruised skin and traumatized muscles, GPA staining revealed RBCs extravasation with interstitial hemorrhage.

In all cases reported in the literature, including those presented in our study, GPA has demonstrated high reliability in severely decomposed bodies, as evidenced by the wide range of PMIs over which significant results have been obtained.

These results provide additional insights beyond the experimental observations of Taborelli et al. [8], who, while noting a temporal superiority of anti-GPA positivity over H&E staining for detecting hemorrhaging RBCs, limited the utility of the glycophorin antigen to a maximum of six days in water and fifteen days in air. The same authors, however, acknowledged that the small skin fragments used and subjected to experimental modifications were not comparable to samples taken from whole bodies, where a longer persistence of antigen survival was assumed.

Through our review, we were able to highlight that GPA validity has been extensively studied throughout the years, in different countries, and with a standardized method of immunohistochemical staining and microscopic evaluation. GPA could be an ideal parameter, as it has been demonstrated that it is absent outside the vessels in non-hemorrhagic conditions and appears regularly after blood extravasation in the tissue. Indicative results came from the analysis of the literature, indicating that GPA in decomposed bodies can be considered a reliable and valid marker of vitality in forensic cases.

There is undoubtedly significant emphasis on research into the reliability and validity of forensic science methods and evidence analysis. Given the critical role that forensic evidence plays in criminal investigations, the judicial scrutiny of the scientific foundations underlying such evidence is of paramount importance. When properly validated and correctly applied, scientific methods can substantially contribute to the pursuit of truth within the criminal justice system.

In the United States, the admissibility of scientific evidence in the Court has been reshaped by the so-called Daubert criteria, which have interpreted the Federal Rules of Evidence, inquiring into the scientific validity of the methodology.

This inquiry looks at four flexible factors [29]:

(a) The validity of the scientific principle or its method;
(b) The correct application of the scientific principle, its technology, and its method;
(c) The specific appropriateness of the scientific principle, its technology, and its method to obtain useful information in the reconstruction of the event;
(d) The data and the facts on which the expert opinion is based need to be recognized by other experts in the field.

The aforementioned criteria were recently applied to evaluate the validity of GPA and, consequently, determine whether its results could be used as scientific evidence in an Italian courtroom. Specifically, an old case was reopened more than 20 years after the first autopsy, and GPA was performed on the specimens to discriminate between vital and non-vital wounds. Interestingly, the lesions initially considered directly related to the cause of death showed no signs of positive staining, suggesting that they must have occurred in the perimortem or even postmortem period. Instead, positivity for GPA was observed in the soft tissues of the neck, particularly at the laryngotracheal level, indicative of violent mechanical asphyxia. Therefore, the cause and manner of death identified by the first autopsy, which had led to the case being classified as a suicide, were no longer considered valid. The substantial evidence obtained during the second round of postmortem investigations led to the initiation of a trial for voluntary manslaughter, which resulted in a conviction (not yet final) [30].

Collectively, the data emerging on GPA from the review of the existing literature demonstrated that the use of GPA could be a very important and reliable determination for forensic experts when they are gathering evidence that can support or decline wound vitality, mostly in decomposed bodies [31].

Ultimately, it is a method that has passed the test of “verification”, meaning the ability to determine the presence or absence of blood material in tissue through GPA tests, which has been proven true through a systematic review of the evidence.

In this regard, it is important to emphasize that in such cases, standardized laboratory methodology should be followed in searching for GPA [32]. In order to achieve optimal results from the immunohistochemistry investigation, a meticulous preparation methodology must be adopted following the protocol outlined below [9,14].

All samples collected during autopsy should be sectioned at 4 μm thickness from paraffin-embedded tissue and stained with H&E, following standard protocols. H&E staining should be qualitatively assessed and classified as “reliable” (++), “not reliable” (–), or “not univocal” (+−), based on the morphological identifiability of RBCs.

Additionally, immunohistochemical analysis should be performed using anti-human GPA antibodies (Santa Cruz Biotechnology, Inc., Dallas, TX, USA). Paraffin sections should be mounted on slides coated with 3-aminopropyltriethoxysilane (Fluka, Buchs, Switzerland). Pre-treatment is essential to facilitate antigen retrieval and enhance membrane permeability to anti-GPA antibodies; this involves boiling with 0.25 M EDTA buffer at 20 °C. The primary antibody should be applied at a 1:500 dilution for GPA and incubated for 120 min at 20 °C.

The detection system employed should be the LSAB+ kit (Dako, Copenhagen, Denmark), a refined avidin–biotin technique wherein a biotinylated secondary antibody interacts with multiple peroxidase-conjugated streptavidin molecules. Sections should then be counterstained with Mayer’s hematoxylin, dehydrated, cover-slipped, and examined using a Leica DM4000B optical microscope (Leica, Cambridge, UK) [9,14].

## 7. Limitations

The primary limitation of the present study is the small number of cases retrieved from the existing literature, which may not represent the general population accurately. Moreover, the absence of recent double-blind case-control studies significantly hinders a robust evaluation of the sensitivity and specificity of anti-GPA antibodies in assessing lesion vitality.

Evidence for GPC is further limited, as only two studies [15,22] employing this methodology were identified, neither of which provide justification for its selection over GPA.

Another important consideration is the potential for both false-negative and false-positive results in glycophorin staining. False negatives may occur due to suboptimal sampling in the absence of a standardized procedure. On the other hand, false positives may arise from immunohistochemical cross-reactivity, as glycophorins can also be detected in tissues rich in erythroid precursor cells, such as bone marrow or sites of extramedullary hematopoiesis.

Furthermore, the assessment of lesion vitality remains closely tied to the correlation with hemorrhagic phenomena in the absence of standardized criteria for histological characterization. In this context, postmortem pseudo-bruising must also be taken into account, when gravitational blood pooling or hypostasis may mimic antemortem injury. Differentiating between true antemortem trauma and postmortem artifacts continues to present a diagnostic challenge in forensic pathology.

## 8. Conclusions

The aim of this study, from a forensic pathology perspective, is to provide scientific support in cases where it is necessary to demonstrate the vitality of a lesion [33,34]. The findings suggest that GPA may serve as a reliable immunohistochemical marker of vitality. Among the various markers evaluated in the literature, GPA has consistently proven to be the most effective. This technique can no longer be regarded as merely experimental, as its application has also been recognized at the judicial level given the reliability of the results. However, it is important to note that this reliability is dependent on the correct operational methodology and the involvement of highly qualified laboratories. Therefore, the methodology presented here may serve as an operational framework for similar cases in the future.

## Data Availability

The datasets generated during and analyzed during the current study are available from the corresponding author on reasonable request.

## Supplementary Materials

The following supporting information can be downloaded at: https://www.mdpi.com/article/10.3390/ijms26115308/s1.

## Abbreviations

The following main abbreviations are used in this manuscript:

GPA	Glycophorin A
GPC	Glycophorin C
H&E	Hematoxylin and eosin
PMI	Postmortem interval
RBC	Red blood cell
