# Supplementary material for: Bloody Evidence: The Validity of Glycophorin A in the Determination of Wound Vitality—A Systematic Review of the Literature
Source: Int J Mol Sci. 2025 May 31;26(11):5308. doi: 10.3390/ijms26115308 (PMC12155515; doi:10.3390/ijms26115308)
Supplement: Supplementary file 1 [file ijms-26-05308-s001.zip › ijms-3606798-supplementary.pdf]

Supplement 1

Appendix SA – Search Strategy

- Research conducted on 27th January 2025.

PUBMED

((Glycophorin* OR Glycophorin A OR Glycophorin B OR Glycophorin C OR Glycophorin D OR Glycophorin E OR Glycophorin HA [Title/Abstract]))

AND

((Autops* OR Death* OR Murder* OR Forensic* OR Forensic Medicine OR Forensic Pathology OR Forensic Science* OR Bleeding* OR Hemorrhage* OR Haemorrhage* OR Wound* OR Vitalit* OR Injur* OR Killing* OR Lesion* OR Decompos* Putrefact*[Title/Abstract]))

Results n = **213**

SCOPUS

(TITLE-ABS-KEY (glycophorin*) OR TITLE-ABS-KEY (glycophorin AND a) OR TITLE-ABS-KEY (glycophorin AND b) OR TITLE-ABS-KEY (glycophorin AND c) OR TITLE-ABS-KEY (glycophorin AND d) OR TITLE-ABS-KEY (glycophorin AND e) OR TITLE-ABS-KEY (glycophorin AND ha) AND TITLE-ABS-KEY (autops*) OR TITLE-ABS-KEY (death*) OR TITLE-ABS-KEY (murder*) OR TITLE-ABS-KEY (forensic*) OR TITLE-ABS-KEY (forensic AND medicine) OR TITLE-ABS-KEY (forensic AND pathology) OR TITLE-ABS-KEY (forensic AND science*) OR TITLE-ABS-KEY (bleeding*) OR TITLE-ABS-KEY (hemorrhage*) OR TITLE-ABS-KEY (haemorrhage*) OR TITLE-ABS-KEY (wound*) OR TITLE-ABS-KEY (vitalit*) OR TITLE-ABS-KEY (injur*) OR TITLE-ABS-KEY (killing*) OR TITLE-ABS-KEY (lesion*) OR TITLE-ABS-KEY (decompos*) OR TITLE-ABS-KEY (putrefact*))

Results n = **386**

WOS

((Glycophorin* OR Glycophorin A OR Glycophorin B OR Glycophorin C OR Glycophorin D OR Glycophorin E OR Glycophorin HA [Title/Abstract]))

AND

((Autops* OR Death* OR Murder* OR Forensic* OR Forensic Medicine OR Forensic Pathology OR Forensic Science* OR Bleeding* OR Hemorrhage* OR Haemorrhage* OR Wound* OR Vitalit* OR Injur* OR Killing* OR Lesion* OR Decompos* Putrefact*[Title/Abstract]))

Results n= **200**

- Sum of the results n = 799

Appendix SB – Screening Process

- Duplicates automatically removed n = 201

- Duplicates manually removed n = 139

- Results screened by titles and abstracts n = 459

- Results screened by full-text n = 50

- Case reports, case series, and case-control studies included n = 10

- Experimental study included n = 2

- Studies for narrative review included n = 4

Appendix SC – List of the Included and Excluded Studies, with motivation

1. Way S, Lachar G, Givens M. A child with fever, malaise, and a skin wound. Pediatr Emerg Care. 2005 Sep;21(9):620-3. doi: 10.1097/01.pec.0000177205.98902.1e. PMID: 16160671.	Wrong focus
2. Kibayashi K, Higashi T, Tsunenari S. A differential study between antemortem bleeding and a postmortem infiltration of hemoglobin. Nihon Hoigaku Zasshi. 1991 Jun;45(3):227-32. PMID: 1920929.	Not English
3. Bowden A, Fleming R, Harbison S. A method for DNA and RNA co-extraction for use on forensic samples using the Promega DNA IQ™ system. Forensic Sci Int Genet. 2011 Jan;5(1):64-8. doi: 10.1016/j.fsigen.2009.11.007. Epub 2010 Jan 6. PMID: 20457058.	Focus on laboratory investigations
4. Castelló A, Francés F, Verdú F. An alternative to the human hemoglobin test in the investigation of bloodstains treated with active oxygen: the human glycophorin A test. ScientificWorldJournal. 2011 Apr 19;11:907-16. doi: 10.1100/tsw.2011.89. PMID: 21516287; PMCID: PMC5720065.	Focus on laboratory investigations
5. Vignali G, Franceschetti L, Attisano GCL, Cattaneo C. Assessing wound vitality in decomposed bodies: a review of the literature. Int J Legal Med. 2023 Mar;137(2):459-470. doi: 10.1007/s00414-022-02932-9. Epub 2022 Dec 23. PMID: 36550324.	Review
6. Seidner, H. S., Gunter, G., Weber-Fishkin, S., & Frame, M. D. (2016). Band 3 and glycophorin C as potential mediators of erythrocyte aggregation during the onset of thermal burn injury. The FASEB Journal, 30, 946-7.	Not available by our institution
7. Gross ML, Meyer HP, Ziebart H, Rieger P, Wenzel U, Amann K, Berger I, Adamczak M, Schirmacher P, Ritz E. Calcification of coronary intima and media: immunohistochemistry, backscatter imaging, and x-ray analysis in renal and nonrenal patients. Clin J Am Soc Nephrol. 2007 Jan;2(1):121-34. doi: 10.2215/CJN.01760506. Epub 2006 Dec 6. PMID: 17699396.	Focus on non-forensic cause of death
8. von der Thüsen JH, van Bommel J, Kros JM, Verdijk RM, Lopuhaä B, Lam KH, Dik WA, Miedema JR. Case report: a fatal combination of hemophagocytic lymphohistiocytosis with extensive pulmonary microvascular damage in	Focus on non-forensic cause of death

COVID-19 pneumonia. J Hematop. 2020 Oct 23;14(1):79-83. doi: 10.1007/s12308-020-00423-7. PMID: 33110452; PMCID: PMC7581498.	
9. Hadjiyannakis A, Fletcher WA, Lebrun DP, van Wylick RC, Dow KE. Congenital erythroleukemia in a neonate with severe hypoxic ischemic encephalopathy. Am J Perinatol. 1998;15(12):689-94. doi: 10.1055/s-2007-999303. PMID: 10333396.	Focus on non-forensic cause of death
10. Vanĕrková H, Klír P, Jezková J, Fedorowiczová A. Průkaz volného histaminu a sérotoninu pri posuzování vitální reakce poranĕní [Detection of free histamine and serotonin in the evaluation of the vital reaction in injuries]. Soud Lek. 1997 Aug;42(3):39-42. Czech. PMID: 9471733.	Not English
11. Frascione N, Pinto V, Daniel B. Development of a biosensor for human blood: new routes to body fluid identification. Anal Bioanal Chem. 2012 Jul;404(1):23-8. doi: 10.1007/s00216-012-6111-7. Epub 2012 May 30. PMID: 22644154.	Focus on laboratory investigations
12. Xu Y, Xie J, Cao Y, Zhou H, Ping Y, Chen L, Gu L, Hu W, Bi G, Ge J, Chen X, Zhao Z. Development of highly sensitive and specific mRNA multiplex system (XCYR1) for forensic human body fluids and tissues identification. PLoS One. 2014 Jul 3;9(7):e100123. doi: 10.1371/journal.pone.0100123. Erratum in: PLoS One. 2014;9(8):e105448. PMID: 24991806; PMCID: PMC4089028.	Focus on laboratory investigations
13. Schweers BA, Old J, Boonlayangoor PW, Reich KA. Developmental validation of a novel lateral flow strip test for rapid identification of human blood (Rapid Stain Identification--Blood). Forensic Sci Int Genet. 2008 Jun;2(3):243-7. doi: 10.1016/j.fsigen.2007.12.006. Epub 2008 Feb 5. PMID: 19083828.	Focus on laboratory investigations
14. Tsunerari S, Kibayashi K, HigashiT. (1991). Differentiation between antemortem bleeding and postmortem infiltration of hemoglobin (Doctoral dissertation, 熊本大学).	Not English
15. Kibayashi K, Hamada K, Honjyo K, Tsunenari S. Differentiation between bruises and putrefactive discolorations of the skin by immunological analysis of glycophorin A. Forensic Sci Int. 1993 Oct;61(2-3):111-7. doi: 10.1016/0379-0738(93)90219-z. PMID: 8307520.	Inlcuded

16. Kibayashi K, Honjyo K, Higashi T, Tsunenari S. Differentiation of discolouration in a body by an erythrocyte membrane component, glycophorin A. Ann Acad Med Singap. 1993 Jan;22(1):28-32. PMID: 8503633.	Not available by our institution
17. Chaturvedi AK, Vu NT, Ritter RM, Canfield DV. DNA typing as a strategy for resolving issues relevant to forensic toxicology. J Forensic Sci. 1999 Jan;44(1):189-92. PMID: 9987885.	Not available by our institution
18. Legaz I, Fineschi V, Madea B, Bacci S. Editorial: Skin lesion vitality assessment for forensic science: Current research and new perspectives. Front Med (Lausanne). 2022 Aug 15;9:969932. doi: 10.3389/fmed.2022.969932. PMID: 36045924; PMCID: PMC9421355.	Editorial
19. Klír P, Vaněrková H, Jezková J. Posuzování vitality vzniku poranění [Evaluation of the vital reaction in injuries]. Soud Lek. 1996 May;41(2):16-9. Czech. PMID: 9560909.	Not English
20. Baldari B, Vittorio S, Sessa F, Cipolloni L, Bertozzi G, Neri M, Cantatore S, Fineschi V, Aromatario M. Forensic Application of Monoclonal Anti-Human Glycophorin A Antibody in Samples from Decomposed Bodies to Establish Vitality of the Injuries. A Preliminary Experimental Study. Healthcare (Basel). 2021 Apr 29;9(5):514. doi: 10.3390/healthcare9050514. PMID: 33946627; PMCID: PMC8145726.	Included
21. Di Fazio N, Scopetti M, Delogu G, Morena D, Santurro A, Cipolloni L, Serviddio G, Papi L, Frati P, Turillazzi E, Fineschi V. Fourteen Deaths from Suspected Heparin Overdose in an Italian Primary-Level Hospital. Diagnostics (Basel). 2023 Nov 1;13(21):3361. doi: 10.3390/diagnostics13213361. PMID: 37958256; PMCID: PMC10650777.	Included (further information not retrieved in the paper obtained directly by the authors)
22. Klír P, Toupalík P, Bouska I, Jezková J. Vyuzití glycophorinu v soudním lékarství [Glycophorin in forensic medicine]. Soud Lek. 2000 May;45(2):22-5. Czech. PMID: 10916933.	Not English
23. Plowey ED, Egbert PR. Histologic Artifacts of Autolytic Müller Cell Foot Process Swelling in Postmortem Examination of Infant Eyes: Potential Pitfall in the Evaluation of Traumatic Retinal Hemorrhages. JAMA Ophthalmol. 2015 Jun;133(6):706-9. doi: 10.1001/jamaophthalmol.2015.0493. PMID: 25837496.	Included

24. Kunio M, Gardecki JA, Watanabe K, Nishimiya K, Verma S, Jaffer FA, Tearney GJ. Histopathological correlation of near infrared autofluorescence in human cadaver coronary arteries. Atherosclerosis. 2022 Mar;344:31-39. doi: 10.1016/j.atherosclerosis.2022.01.012. Epub 2022 Jan 29. PMID: 35134654; PMCID: PMC9106423.	Focus on non-forensic cause of death
25. de Beijer RP, de Graaf C, van Weert A, van Leeuwen TG, Aalders MCG, van Dam A. Identification and detection of protein markers to differentiate between forensically relevant body fluids. Forensic Sci Int. 2018 Sep;290:196-206. doi: 10.1016/j.forsciint.2018.07.013. Epub 2018 Jul 24. PMID: 30071450.	Focus on laboratory investigations
26. Ambrosetti, F., Palazzo, E., Gibelli, D. *et al.* The risk of misinterpreting genital signs of sexual abuse in cadavers: a case report. *Int J Legal Med* 127, 907–910 (2013). https://doi.org/10.1007/s00414-013-0891-y	Included
27. Bertozzi G, Ferrara M, La Russa R, Pollice G, Gurgoglione G, Frisoni P, Alfieri L, De Simone S, Neri M, Cipolloni L. Wound Vitality in Decomposed Bodies: New Frontiers Through Immunohistochemistry. Front Med (Lausanne). 2021 Dec 24;8:802841. doi: 10.3389/fmed.2021.802841. PMID: 35004780; PMCID: PMC8739223.	Included (further information not retrieved in the paper obtained directly by the authors)
28. Cappella A, Bertoglio B, Castoldi E, Maderna E, Di Giancamillo A, Domeneghini C, Andreola S, Cattaneo C. The taphonomy of blood components in decomposing bone and its relevance to physical anthropology. Am J Phys Anthropol. 2015 Dec;158(4):636-45. doi: 10.1002/ajpa.22830. Epub 2015 Aug 12. PMID: 26265488.	Included (only real cases, not experimental ones)
29. Cattaneo, Cristina PhD, MD*; Andreola, Salvatore MD†; Marinelli, Eloisa MD*; Poppa, Pasquale BSc*; Porta, Davide BSc*; Grandi, Marco MD*. The Detection of Microscopic Markers of Hemorrhaging and Wound Age on Dry Bone: A Pilot Study. The American Journal of Forensic Medicine and Pathology 31(1):p 22-26, March 2010. | DOI: 10.1097/PAF.0b013e3181c15d74	Included (Insufficient data for cases #1, #4, #5, and for the #control)

30. Luigi Crudele GD, Galante N, Fociani P, Del Gobbo A, Tambuzzi S, Gentile G, Zoja R. The forensic application of the Glycophorin A on the Amussat's sign with a brief review of the literature. J Forensic Leg Med. 2021 Aug;82:102228. doi: 10.1016/j.jflm.2021.102228. Epub 2021 Aug 10. PMID: 34388443.	Included
31. Cuttaia, C.; Di Stefano, B.; Sorçaburu Ciglieri, S.; Vetrini, R.; Previderè, C.; Fattorini, P. Immunochromatographic Detection of Human Blood: A Forensic Review. *Separations* 2024, *11*, 66. https://doi.org/10.3390/separations11030066	Focus on laboratory investigations
32. Nicola Galante, Stefano Tambuzzi, Guendalina Gentile, Riccardo Zoja, Letter to the Editor regarding the article "Neck vascular lesions in hanging cases: A literature review", Journal of Forensic and Legal Medicine, Volume 85, 2022, 102295, ISSN 1752-928X, https://doi.org/10.1016/j.jflm.2021.102295.<br>(https://www.sciencedirect.com/science/article/pii/S1752928X21001803)	Comment
33. Gauchotte G, Bochnakian A, Campoli P, Lardenois E, Brix M, Simon E, Colomb S, Martrille L, Peyron PA. Myeloperoxydase and CD15 With Glycophorin C Double Staining in the Evaluation of Skin Wound Vitality in Forensic Practice. Front Med (Lausanne). 2022 May 17;9:910093. doi: 10.3389/fmed.2022.910093. PMID: 35665361; PMCID: PMC9156797.	Included in narrative review
34. Ikemoto S. [Searching for genetic markers--in the fields of forensic medicine and human genetics]. Nihon Hoigaku Zasshi. 1995 Dec;49(6):419-31. Japanese. PMID: 8583686.	Not English
35. Aniello Maiese, Serenella Serinelli, Lorenzo Gitto, Pietro Falco, Valeria Panebianco, Giorgio Bolino, The usefulness of post-mortem computed tomography in a crush asphyxia. An excessive enjoyed rave party resulting in a fatal sleep!,	Included

Journal of Forensic Radiology and Imaging, Volume 3, Issue 1, 2015, Pages 91-95,ISSN 2212-4780,https://doi.org/10.1016/j.jofri.2014.12.007.	
36. Debora Mazzarelli, Stefano Tambuzzi, Emanuela Maderna, Giulia Caccia, Pasquale Poppa, Vera Merelli, Marco Terzi, Agostino Rizzi, Luca Trombino, Salvatore Andreola, Cristina Cattaneo, Look before washing and cleaning: A caveat to pathologists and anthropologists, Journal of Forensic and Legal Medicine, Volume 79, 2021, 102137, ISSN 1752-928X, https://doi.org/10.1016/j.jflm.2021.102137.	Included
37. Núñez-Torrón C, Ferrer-Gómez A, Moreno Moreno E, et al Secondary haemophagocytic lymphohistiocytosis in COVID-19: correlation of the autopsy findings of bone marrow haemophagocytosis with HScoreJournal of Clinical Pathology 2022;75:383-389.	Focus on non-forensic cause of death
38. Olds K, Byard RW, Winskog C, Langlois NE. Validation of alternate light sources for detection of bruises in non-embalmed and embalmed cadavers. Forensic Sci Med Pathol. 2017 Mar;13(1):28-33. doi: 10.1007/s12024-016-9822-9. Epub 2016 Dec 26. PMID: 28019003.	Included in the narrative review
39. Cecilia Salzillo, Liliana Innamorato, Alessia Leggio, Andrea Marzullo, Immunohistochemical markers in the determination of lesion viability in decomposed bodies: A mini literature review, Forensic Science International, Volume 365, 2024, 112289, ISSN 0379-0738, https://doi.org/10.1016/j.forsciint.2024.112289.	Review
40. Santurro A, Vullo AM, Borro M, Gentile G, La Russa R, Simmaco M, Frati P, Fineschi V. Personalized Medicine Applied to Forensic Sciences: New Advances and Perspectives for a Tailored Forensic Approach. Curr Pharm Biotechnol. 2017;18(3):263-273. doi: 10.2174/1389201018666170207141525. PMID: 28176637.	Review

41. Yuichiro Sato, Hatsue Ishibashi-Ueda, Takashi Iwakiri, Yoshihiko Ikeda, Takaaki Matsuyama, Kinta Hatakeyama, Yujiro Asada, Thrombus components in cardioembolic and atherothrombotic strokes, Thrombosis Research, Volume 130, Issue 2, 2012, Pages 278-280, ISSN 0049-3848, https://doi.org/10.1016/j.thromres.2012.04.008.	Focus on non-forensic cause of death
42. N Tabata, M Morita, Immunohistochemical demonstration of bleeding in decomposed bodies by using anti-glycophorin A monoclonal antibody, Forensic Science International, Volume 87, Issue 1, 1997, Pages 1-8, ISSN 0379-0738, https://doi.org/10.1016/S0379-0738(97)02118-X.	Included
43. N Tabata, Morphological changes in traumatized skeletal muscle:: The appearance of `opaque fibers' of cervical muscles as evidence of compression to the neck, Forensic Science International, Volume 96, Issues 2–3, 1998, Pages 197-214, ISSN 0379-0738, https://doi.org/10.1016/S0379-0738(98)00125-X.	Included in the narrative review
44. Taborelli A, Andreola S, Di Giancamillo A, Gentile G, Domeneghini C, Grandi M, Cattaneo C. The use of the anti-Glycophorin A antibody in the detection of red blood cell residues in human soft tissue lesions decomposed in air and water: a pilot study. Med Sci Law. 2011;51 Suppl 1:S16-9. doi: 10.1258/msl.2010.010107. PMID: 22021627.	Included in the narrative review
45. Toma, L., Vignali, G., Maffioli, E. et al. Mass spectrometry-based proteomic strategy for ecchymotic skin examination in forensic pathology. Sci Rep 13, 6116 (2023). https://doi.org/10.1038/s41598-023-32520-9	Included in the narrative review
46. Toupalík P, Klír P, Bouska I, Chadová L. Imunohistochemické metody v diferenciální diagnostice primárních úrazových a následných sekundárních změn mozku [Immunohistochemical methods in the differential diagnosis of primary traumatic and subsequent secondary cerebral changes]. Soud	Not English

Lek. 2000 May;45(2):18-21. Czech. PMID: 10916932.	
47. Tsunenari S. [Practice and research of forensic medicine learned from the dead]. Nihon Hoigaku Zasshi. 1998 Oct;52(5):277-85. Japanese. PMID: 10077972.	Not English
48. Stefania Turrina, Giulia Filippini, Renzo Atzei, Elisabetta Zaglia, Domenico De Leo, Validation studies of rapid stain identification-blood (RSID-blood) kit in forensic caseworks, Forensic Science International: Genetics Supplement Series, Volume 1, Issue 1, 2008, Pages 74-75, ISSN 1875-1768, https://doi.org/10.1016/j.fsigss.2007.10.166.	Focus on laboratory investigations
49.Wei Y, Wang J, Wang Q, Cong B, Li S. The estimation of bloodstain age utilizing circRNAs and mRNAs biomarkers. Forensic Sci Int. 2022 Sep;338:111408. doi: 10.1016/j.forsciint.2022.111408. Epub 2022 Jul 22. PMID: 35901585.	Focus on laboratory investigations
50. Zhao H, Wang C, Yao L, Lin Q, Xu X, Hu L, Li W. Identification of aged bloodstains through mRNA profiling: Experiments results on selected markers of 30- and 50-year-old samples. Forensic Sci Int. 2017 Mar;272:e1-e6. doi: 10.1016/j.forsciint.2017.01.006. Epub 2017 Jan 16. PMID: 28122679.	Focus on laboratory investigations

Appendix SD – Risk of Bias

Risk of bias was assessed using JBI's critical appraisal tools (e.g., JBI Critical Appraisal Checklist for Case Reports and JBI Critical Appraisal Checklist for Case Series; see Appendix D in Supplement 1).

Baldari et al. (JBI Critical Appraisal Checklist for Case Series)[2]

	Yes	No	Unclear	Not applicable
• Were there clear criteria for inclusion in the case series?	X	☐	☐	☐
• Was the condition measured in a standard, reliable way for all participants included in the case series?	X	☐	☐	☐
• Were valid methods used for identification of the condition for all participants included in the case series?	X	☐	☐	☐
• Did the case series have consecutive inclusion of participants?	☐	X	☐	☐
• Did the case series have complete inclusion of participants?	X	☐	☐	☐
• Was there clear reporting of the demographics of the participants in the study?	X	☐	☐	☐
• Was there clear reporting of clinical information of the participants?	☐	☐	☐	X
• Were the outcomes or follow up results of cases clearly reported?	☐	☐	☐	X
• Was there clear reporting of the presenting site(s)/clinic(s) demographic information?	X	☐	☐	☐
• Was statistical analysis appropriate?	☐	☐	☐	X

Overall appraisal: Include X Exclude ☐ Seek further info ☐

Comments (Including reason for exclusion)

This study met the inclusion criteria established by our systematic review.

Di Fazio et al. (JBI Critical Appraisal Checklist for Case Series)[2]

	Yes	No	Unclear	Not applicable
• Were there clear criteria for inclusion in the case series?	X	☐	☐	☐
• Was the condition measured in a standard, reliable way for all participants included in the case series?	X	☐	☐	☐
• Were valid methods used for identification of the condition for all participants included in the case series?	X	☐	☐	☐
• Did the case series have consecutive inclusion of participants?	☐	X	☐	☐
• Did the case series have complete inclusion of participants?	X	☐	☐	☐
• Was there clear reporting of the demographics of the participants in the study?	☐	☐	X	☐
• Was there clear reporting of clinical information of the participants?	X	☐	☐	☐
• Were the outcomes or follow up results of cases clearly reported?	☐	☐	☐	X
• Was there clear reporting of the presenting site(s)/clinic(s) demographic information?	X	☐	☐	☐
• Was statistical analysis appropriate?	☐	☐	X	☐

Overall appraisal: Include X Exclude ☐ Seek further info ☐

Comments (Including reason for exclusion)

This study met the inclusion criteria established by our systematic review.

Plowey and Egbert (JBI Critical Appraisal Checklist for Case Series)[2]

	Yes	No	Unclear	Not applicable
• Were there clear criteria for inclusion in the case series?	X	☐	☐	☐
• Was the condition measured in a standard, reliable way for all participants included in the case series?	X	☐	☐	☐
• Were valid methods used for identification of the condition for all participants included in the case series?	☐	X	☐	☐
• Did the case series have consecutive inclusion of participants?	☐	X	☐	☐
• Did the case series have complete inclusion of participants?	☐	☐	X	☐
• Was there clear reporting of the demographics of the participants in the study?	☐	☐	X	☐
• Was there clear reporting of clinical information of the participants?	☐	☐	X	☐
• Were the outcomes or follow up results of cases clearly reported?	☐	☐	☐	X
• Was there clear reporting of the presenting site(s)/clinic(s) demographic information?	☐	☐	X	☐
• Was statistical analysis appropriate?	☐	☐	☐	X

Overall appraisal: Include X Exclude ☐ Seek further info ☐

Comments (Including reason for exclusion)

This study met the inclusion criteria established by our systematic review.

Ambrosetti et al. (JBI CRITICAL APPRAISAL CHECKLIST FOR CASE REPORTS)[1]

	Yes	No	Unclear	Not applicable
1. Were patient's demographic characteristics clearly described?	X	☐	☐	☐
2. Was the patient's history clearly described and presented as a timeline?	X	☐	☐	☐
3. Was the current clinical condition of the patient on presentation clearly described?	X	☐	☐	☐
4. Were diagnostic tests or assessment methods and the results clearly described?	X	☐	☐	☐
5. Was the intervention(s) or treatment procedure(s) clearly described?	☐	☐	☐	X
6. Was the post-intervention clinical condition clearly described?	☐	☐	☐	X
7. Were adverse events (harms) or unanticipated events identified and described?	☐	☐	☐	X
8. Does the case report provide takeaway lessons?	X	☐	☐	☐

Overall appraisal: Include X Exclude ☐ Seek further info ☐

Comments (Including reason for exclusion)

This study met the inclusion criteria established by our systematic review.

Bertozzi et al. (JBI Critical Appraisal Checklist for Case Series)[2]

	Yes	No	Unclear	Not applicable
• Were there clear criteria for inclusion in the case series?	X	☐	☐	☐
• Was the condition measured in a standard, reliable way for all participants included in the case series?	X	☐	☐	☐
• Were valid methods used for identification of the condition for all participants included in the case series?	X	☐	☐	☐
• Did the case series have consecutive inclusion of participants?	☐	X	☐	☐
• Did the case series have complete inclusion of participants?	X	☐	☐	☐
• Was there clear reporting of the demographics of the participants in the study?	☐	X	☐	☐
• Was there clear reporting of clinical information of the participants?	☐	☐	☐	X
• Were the outcomes or follow up results of cases clearly reported?	☐	☐	☐	X
• Was there clear reporting of the presenting site(s)/clinic(s) demographic information?	X	☐	☐	☐
• Was statistical analysis appropriate?	☐	☐	☐	X

Overall appraisal: Include X Exclude ☐ Seek further info ☐

Comments (Including reason for exclusion)

This study met the inclusion criteria established by our systematic review.

Cattaneo et al. (JBI Critical Appraisal Checklist for Case Series)[2]

	Yes	No	Unclear	Not applicable
• Were there clear criteria for inclusion in the case series?	X	☐	☐	☐
• Was the condition measured in a standard, reliable way for all participants included in the case series?	X	☐	☐	☐
• Were valid methods used for identification of the condition for all participants included in the case series?	X	☐	☐	☐
• Did the case series have consecutive inclusion of participants?	☐	X	☐	☐
• Did the case series have complete inclusion of participants?	X	☐	☐	☐
• Was there clear reporting of the demographics of the participants in the study?	☐	☐	☐	X
• Was there clear reporting of clinical information of the participants?	☐	☐	☐	X
• Were the outcomes or follow up results of cases clearly reported?	☐	☐	☐	X
• Was there clear reporting of the presenting site(s)/clinic(s) demographic information?	X	☐	☐	☐
• Was statistical analysis appropriate?	☐	☐	☐	X

Overall appraisal: Include X Exclude ☐ Seek further info ☐

Comments (Including reason for exclusion)

This study met the inclusion criteria established by our systematic review.

Crudele et al. (JBI CRITICAL APPRAISAL CHECKLIST FOR CASE REPORTS) [1]

	Yes	No	Unclear	Not applicable
9. Were patient's demographic characteristics clearly described?	X	☐	☐	☐
10. Was the patient's history clearly described and presented as a timeline?	X	☐	☐	☐
11. Was the current clinical condition of the patient on presentation clearly described?	X	☐	☐	☐
12. Were diagnostic tests or assessment methods and the results clearly described?	X	☐	☐	☐
13. Was the intervention(s) or treatment procedure(s) clearly described?	☐	☐	☐	X
14. Was the post-intervention clinical condition clearly described?	☐	☐	☐	X
15. Were adverse events (harms) or unanticipated events identified and described?	☐	☐	☐	X
16. Does the case report provide takeaway lessons?	X	☐	☐	☐

Overall appraisal: Include X Exclude ☐ Seek further info ☐

Comments (Including reason for exclusion)

This study met the inclusion criteria established by our systematic review.

Maiese et al. (JBI CRITICAL APPRAISAL CHECKLIST FOR CASE REPORTS) [1]

	Yes	No	Unclear	Not applicable
17. Were patient's demographic characteristics clearly described?	X	☐	☐	☐
18. Was the patient's history clearly described and presented as a timeline?	X	☐	☐	☐
19. Was the current clinical condition of the patient on presentation clearly described?	X	☐	☐	☐
20. Were diagnostic tests or assessment methods and the results clearly described?	X	☐	☐	☐
21. Was the intervention(s) or treatment procedure(s) clearly described?	☐	☐	☐	X
22. Was the post-intervention clinical condition clearly described?	☐	☐	☐	X
23. Were adverse events (harms) or unanticipated events identified and described?	☐	☐	☐	X
24. Does the case report provide takeaway lessons?	X	☐	☐	☐

Overall appraisal: Include X Exclude ☐ Seek further info ☐

Comments (Including reason for exclusion)

This study met the inclusion criteria established by our systematic review.

Mazzarelli et al. (JBI CRITICAL APPRAISAL CHECKLIST FOR CASE REPORTS)[1]

	Yes	No	Unclear	Not applicable
25. Were patient's demographic characteristics clearly described?	☐	☐	X	☐
26. Was the patient's history clearly described and presented as a timeline?	X	☐	☐	☐
27. Was the current clinical condition of the patient on presentation clearly described?	X	☐	☐	☐
28. Were diagnostic tests or assessment methods and the results clearly described?	X	☐	☐	☐
29. Was the intervention(s) or treatment procedure(s) clearly described?	☐	☐	☐	X
30. Was the post-intervention clinical condition clearly described?	☐	☐	☐	X
31. Were adverse events (harms) or unanticipated events identified and described?	☐	☐	☐	X
32. Does the case report provide takeaway lessons?	X	☐	☐	☐

Overall appraisal: Include X Exclude ☐ Seek further info ☐

Comments (Including reason for exclusion)

This study met the inclusion criteria established by our systematic review.

Tabata and Morita (JBI Critical Appraisal Checklist for Case Series)[2]

	Yes	No	Unclear	Not applicable
• Were there clear criteria for inclusion in the case series?	☐	☐	X	☐
• Was the condition measured in a standard, reliable way for all participants included in the case series?	X	☐	☐	☐
• Were valid methods used for identification of the condition for all participants included in the case series?	X	☐	☐	☐
• Did the case series have consecutive inclusion of participants?	☐	X	☐	☐
• Did the case series have complete inclusion of participants?	X	☐	☐	☐
• Was there clear reporting of the demographics of the participants in the study?	☐	☐	☐	X
• Was there clear reporting of clinical information of the participants?	☐	☐	☐	X
• Were the outcomes or follow up results of cases clearly reported?	☐	☐	☐	X
• Was there clear reporting of the presenting site(s)/clinic(s) demographic information?	X	☐	☐	☐
• Was statistical analysis appropriate?	☐	☐	☐	X

Overall appraisal: Include X Exclude ☐ Seek further info ☐

Comments (Including reason for exclusion)

This study met the inclusion criteria established by our systematic review.

Cappella et al. (JBI Critical Appraisal Checklist for Case Series)[2]

	Yes	No	Unclear	Not applicable
• Were there clear criteria for inclusion in the case series?	☐	X	☐	☐
• Was the condition measured in a standard, reliable way for all participants included in the case series?	X	☐	☐	☐
• Were valid methods used for identification of the condition for all participants included in the case series?	X	☐	☐	☐
• Did the case series have consecutive inclusion of participants?	☐	X	☐	☐
• Did the case series have complete inclusion of participants?	X	☐	☐	☐
• Was there clear reporting of the demographics of the participants in the study?	☐	☐	☐	X
• Was there clear reporting of clinical information of the participants?	☐	☐	☐	X
• Were the outcomes or follow up results of cases clearly reported?	☐	☐	☐	X
• Was there clear reporting of the presenting site(s)/clinic(s) demographic information?	X	☐	☐	☐
• Was statistical analysis appropriate?	☐	☐	☐	X

Overall appraisal: Include X Exclude ☐ Seek further info ☐

Comments (Including reason for exclusion)

This study met the inclusion criteria established by our systematic review.

Kibayashi et al. (JBI Critical Appraisal Checklist for Case Control Studies)

	Yes	No	Unclear	Not applicable
1. Were the groups comparable other than the presence of disease in cases or the absence of disease in controls?	☐	☐	☐	x
2. Were cases and controls matched appropriately?	☐	☐	☐	x
3. Were the same criteria used for identification of cases and controls?	x	☐	☐	☐
4. Was exposure measured in a standard, valid and reliable way?	x	☐	☐	☐
5. Was exposure measured in the same way for cases and controls?	x	☐	☐	☐
6. Were confounding factors identified?	x	☐	☐	☐
7. Were strategies to deal with confounding factors stated?	☐	☐	☐	x
8. Were outcomes assessed in a standard, valid and reliable way for cases and controls?	x	☐	☐	☐
9. Was the exposure period of interest long enough to be meaningful?	☐	☐	☐	x
10. Was appropriate statistical analysis used?	☐	☐	x	☐

Overall appraisal: Include x Exclude ☐ Seek further info ☐

Comments (Including reason for exclusion)

This study met the inclusion criteria established by our systematic review.

Taborelli et al. (JBI Critical Appraisal Checklist for Case Control Studies)

	Yes	No	Unclear	Not applicable
1. Were the groups comparable other than the presence of disease in cases or the absence of disease in controls?	☐	☐	☐	X
2. Were cases and controls matched appropriately?	X	☐	☐	☐
3. Were the same criteria used for identification of cases and controls?	X	☐	☐	☐
4. Was exposure measured in a standard, valid and reliable way?	X	☐	☐	☐
5. Was exposure measured in the same way for cases and controls?	X	☐	☐	☐
6. Were confounding factors identified?	☐	☐	X	☐
7. Were strategies to deal with confounding factors stated?	☐	☐	X	☐
8. Were outcomes assessed in a standard, valid and reliable way for cases and controls?	X	☐	☐	☐
9. Was the exposure period of interest long enough to be meaningful?	X	☐	☐	☐
10. Was appropriate statistical analysis used?	☐	☐	☐	X

Overall appraisal: Include X Exclude ☐ Seek further info ☐

Comments (Including reason for exclusion)

This study met the inclusion criteria established by our systematic review.

References

- Moola, S., Munn, Z., Tufanaru, C., Aromataris, E., Sears, K., Sfetcu, R., Currie, M., Qureshi, R., Mattis, P., Lisy, K., Mu, P-F. Chapter 7: Systematic reviews of etiology and risk. In: JBI Manual for Evidence Synthesis; Aromataris, E., Munn, Z., Eds; JBI, 2020.

- Munn, Z., Barker, T.H., Moola, S., Tufanaru, C., Stern, C., McArthur, A., Stephenson, M., Aromataris, E. Methodological quali-ty of case series studies: an introduction to the JBI critical appraisal tool. JBI Evid Synth. 2020, 10, 2127-2133.

PRISMA 2020 Main Checklist

Topic	No.	Item	Location where item is reported
**TITLE**			
**Title**	1	Identify the report as a systematic review.	Line 3, Page 1
**ABSTRACT**			
**Abstract**	2	See the PRISMA 2020 for Abstracts checklist	Page 1
**INTRODUCTION**			
**Rationale**	3	Describe the rationale for the review in the context of existing knowledge.	Section 1, Page 2
**Objectives**	4	Provide an explicit statement of the objective(s) or question(s) the review addresses.	Section 1, Page 3
**METHODS**			
**Eligibility criteria**	5	Specify the inclusion and exclusion criteria for the review and how studies were grouped for the syntheses.	Section 2, Page 3
**Information sources**	6	Specify all databases, registers, websites, organisations, reference lists and other sources searched or consulted to identify studies. Specify the date when each source was last searched or consulted.	Section 2, Page 3
**Search strategy**	7	Present the full search strategies for all databases, registers and websites, including any filters and limits used.	Appendix A
**Selection process**	8	Specify the methods used to decide whether a study met the inclusion criteria of the review, including how many reviewers screened each record and each report retrieved, whether they worked independently, and if applicable, details of automation tools used in the process.	Section 2, Page 3

Topic	No.	Item	Location where item is reported
**Data collection process**	9	Specify the methods used to collect data from reports, including how many reviewers collected data from each report, whether they worked independently, any processes for obtaining or confirming data from study investigators, and if applicable, details of automation tools used in the process.	Section 2, Page 3
**Data items**	10a	List and define all outcomes for which data were sought. Specify whether all results that were compatible with each outcome domain in each study were sought (e.g. for all measures, time points, analyses), and if not, the methods used to decide which results to collect.	Section 3, Page 3
	10b	List and define all other variables for which data were sought (e.g. participant and intervention characteristics, funding sources). Describe any assumptions made about any missing or unclear information.	Section 3, Page 3
**Study risk of bias assessment**	11	Specify the methods used to assess risk of bias in the included studies, including details of the tool(s) used, how many reviewers assessed each study and whether they worked independently, and if applicable, details of automation tools used in the process.	NA
**Effect measures**	12	Specify for each outcome the effect measure(s) (e.g. risk ratio, mean difference) used in the synthesis or presentation of results.	NA
**Synthesis methods**	13a	Describe the processes used to decide which studies were eligible for each synthesis (e.g. tabulating the study intervention characteristics and comparing against the planned groups for each synthesis (item 5)).	Table 1
	13b	Describe any methods required to prepare the data for presentation or synthesis, such as handling of missing summary statistics, or data conversions.	Table 1
	13c	Describe any methods used to tabulate or visually display results of individual studies and syntheses.	Table 1

Topic	No.	Item	Location where item is reported
	13d	Describe any methods used to synthesize results and provide a rationale for the choice(s). If meta-analysis was performed, describe the model(s), method(s) to identify the presence and extent of statistical heterogeneity, and software package(s) used.	Table 1
	13e	Describe any methods used to explore possible causes of heterogeneity among study results (e.g. subgroup analysis, meta-regression).	NA
	13f	Describe any sensitivity analyses conducted to assess robustness of the synthesized results.	NA
**Reporting bias assessment**	14	Describe any methods used to assess risk of bias due to missing results in a synthesis (arising from reporting biases).	NA
**Certainty assessment**	15	Describe any methods used to assess certainty (or confidence) in the body of evidence for an outcome.	NA
**RESULTS**			
**Study selection**	16a	Describe the results of the search and selection process, from the number of records identified in the search to the number of studies included in the review, ideally using a flow diagram.	Figure 3, Section 4, Page 4
	16b	Cite studies that might appear to meet the inclusion criteria, but which were excluded, and explain why they were excluded.	Appendix C
**Study characteristics**	17	Cite each included study and present its characteristics.	Table 1
**Risk of bias in studies**	18	Present assessments of risk of bias for each included study.	NA
**Results of individual studies**	19	For all outcomes, present, for each study: (a) summary statistics for each group (where appropriate) and (b) an effect estimate and its precision (e.g. confidence/credible interval), ideally using structured tables or plots.	Tabe 1, Figure 4 and 5

Topic	No.	Item	Location where item is reported
**Results of syntheses**	20a	For each synthesis, briefly summarise the characteristics and risk of bias among contributing studies.	NA
	20b	Present results of all statistical syntheses conducted. If meta-analysis was done, present for each the summary estimate and its precision (e.g. confidence/credible interval) and measures of statistical heterogeneity. If comparing groups, describe the direction of the effect.	Section 5, Page 12
	20c	Present results of all investigations of possible causes of heterogeneity among study results.	NA
	20d	Present results of all sensitivity analyses conducted to assess the robustness of the synthesized results.	NA
**Reporting biases**	21	Present assessments of risk of bias due to missing results (arising from reporting biases) for each synthesis assessed.	NA
**Certainty of evidence**	22	Present assessments of certainty (or confidence) in the body of evidence for each outcome assessed.	NA
**DISCUSSION**			
**Discussion**	23a	Provide a general interpretation of the results in the context of other evidence.	Section 6, Page 12-14
	23b	Discuss any limitations of the evidence included in the review.	Section 7, Page 14
	23c	Discuss any limitations of the review processes used.	NA
	23d	Discuss implications of the results for practice, policy, and future research.	Section 8, Page 15
**OTHER INFORMATION**			

Topic	No.	Item	Location where item is reported
**Registration and protocol**	24a	Provide registration information for the review, including register name and registration number, or state that the review was not registered.	NA
	24b	Indicate where the review protocol can be accessed, or state that a protocol was not prepared.	NA
	24c	Describe and explain any amendments to information provided at registration or in the protocol.	NA
**Support**	25	Describe sources of financial or non-financial support for the review, and the role of the funders or sponsors in the review.	Page 15
**Competing interests**	26	Declare any competing interests of review authors.	Page 15
**Availability of data, code and other materials**	27	Report which of the following are publicly available and where they can be found: template data collection forms; data extracted from included studies; data used for all analyses; analytic code; any other materials used in the review.	NA

From: Page MJ, McKenzie JE, Bossuyt PM, Boutron I, Hoffmann TC, Mulrow CD, et al. The PRISMA 2020 statement: an updated guideline for reporting systematic reviews. MetaArXiv. 2020, September 14. DOI: 10.31222/osf.io/v7gm2. For more information, visit: www.prisma-statement.org
